# Supplementary material for: Development and application of the Demands for Population Health Interventions (Depth) framework for categorising the agentic demands of population health interventions
Source: BMC Glob Public Health. 2024 Mar 4;2:13. doi: 10.1186/s44263-024-00043-8 (PMC11622985; doi:10.1186/s44263-024-00043-8)
Supplement: Supplementary file 3 — Additional file 3: Details of interventions included in testing the DePtH framework. Table S11. Table of study characteristics. [file 44263_2024_43_MOESM3_ESM.docx]

**Additional File 3 – Details of interventions included in testing the DePtH framework**

| **Table S11 -** *Table of study characteristics* | | | | | | | |
| --- | --- | --- | --- | --- | --- | --- | --- |
| **Study design, aim** | **Participants/ population** | **Behavioural target** | **Intervention summary** | **Intervention components-recipient combinations (n)** | **Effectiveness measure and category** | **SEP Measure and category** | **Quality rating ^a^** |
| **Bere-2005 (Norway)(1)** | | | | | | | |
| Design: Repeat cross-sectional with unexposed group  Aim: To investigate the effect of free fruit provision at school on the consumption of fruit and vegetables and ‘unhealthy’ snacks | 7^th^ grade pupils from 29 schools in Hedmark and Telemark counties in Norway (n=638) | Diet | Pupils receive a piece of fruit or carrot each school day in connection with their lunch meal, provided for free. | Recipients: School children  Components (n=1)  1.Free school fruit | Fruit and vegetable intake at school  Results favour intervention | Parent education level  No preferential impact | Moderate |
| **Bere-2010 (Norway)(2)** | | | | | | | |
| Design: Repeat cross-sectional with unexposed group  Aim: Evaluate nationwide implementation of free school fruit on adolescent fruit and vegetable intake | 6^th^ and 7^th^ grade pupils from 17 schools in Hedmark and Telemark counties | Diet | Official free school fruit programme where children are entitled to a free piece of fruit or vegetable every school day | Recipients: School children  Components (n=1)  1.Free school fruit | Fruit and vegetable intake at school  Results favour intervention | Parental education level  No preferential impact | Weak |
| **Brownson-1996 (USA)(3)** | | | | | | | |
| Design: Repeat cross-sectional  Aim: Evaluate impact of the Bootheel Heart Health Project on cardiovascular disease risk factors | Representative sample of adults in six counties in southeastern Missouri. Region characterised by high rates of poverty and low education levels | Diet and PA | Six community coalitions receive $5000 per year to select, prioritise and implement community based interventions. | Recipients: Adults in Botheel region  Components (n=11)  1. Walking clubs  2. Aerobic exercise classes  3. Community blood pressure, diabetes and cholesterol screening  4. Cardiovascular disease education programs  5. Heart Healthy Cooking Demonstrations  6. Exercise demonstrations  7. Heart disease education in sermons  8. Heart Healthy Dinners served in church  9. Poster contest  10. Weekly newspaper column  11. Environmental changes, eg, walking path | Prevalence of 5+ serving fruit and vegetable daily  Diet: No difference  PA: Results favour intervention | Education level  Diet: Likely to reduce inequalities  PA: No preferential impact | 3 |
| **Campbell-1999 (USA)(4)** | | | | | | | |
| Design: RCT  Aim: To assess effectiveness of the intervention to increase fruit and vegetable intake among rural African American Church Members | African American attendees of churches in 10 rural counties in eastern North Carolina (n=2,519) | Diet | Multi-component intervention targeting pre-disposing factors, enabling factors and reinforcing factors | Recipients: Members of church  Components (n=18)  1. Tailored bulletins  2. Printed materials – brochures  3. Printed materials – posters  4. Printed materials – banners  5. Printed materials – bulletin board materials  6. Printed materials – idea sheets  7. Printed materials – church bulletin inserts  8. Gardening  9. Educational sessions on cooking  10. Cookbook and recipe tasting  11. Serving more fruit and vegetables at church functions  12. Lay health advisors  13. Community coalitions to plan community events  14. Pastor support  15. Grocer-vendor involvement – farmers market posters  16. Grocer-vendor involvement – coupon  17. Grocer-vendor involvement – recipe cards  18. Church initiated events | Fruit and vegetable consumption  Results favour intervention | Household income  No preferential impact | 5 |
| **Coronini Cronberg-2012 (UK)(5)** | | | | | | | |
| Design: Repeat cross-sectional  Aim: To assess the impact of a free older persons’ bus pass on active travel and regular walking in England | Adults aged over 60 in England  (n=16,911) | PA | Free bus pass for older adults | Recipients: Adults aged 60+  Components (n=1)  1. Free bus pass | Use of active transport, buses and walking 3 or more times/week  Results favour intervention | Housing tenure  No preferential impact | Moderate |
| **Cullen-2009 & Mendoza-2010 (USA)(6-8)** | | | | | | | |
| Design: Repeat cross-sectional  Aim: Compare impact of Texas Public School Nutrition Policy on lunch consumption of low and middle income students | Two schools in southeast Texas | Diet | An unfunded mandate to promote healthy school environments restricting portion sizes of snack and high fat foods, sales of sweetened beverages and the fat content of all foods. | Recipients: School children  Components (n=5)  1.Restrict portion sizes of sugar-sweetened beverages in schools  2. Limit frequency and serving size of high fat vegetables  3. Restrict portion sizes of snacks and high fat foods  4. Restricts sales of sweetened beverages  5. Restricted fat content of all foods | Nutrient, food group and energy density consumption  Results favour intervention | School level free school meals  No preferential impact | Weak |
| **Fogarty-2007 (UK)(9)** | | | | | | | |
| Design: Repeat cross-sectional study  Aim: Determine effect of school fruit provision of fruit intake in young children and any effect after intervention ceased | 4-6 year old children attending 113 local authority maintained schools in East Midlands, England | Diet | National Schools Fruit Scheme provided a daily piece of fruit distributed at school | Recipients: School children  Components (n=1)  1. Provision of free fruit at school | Fruit consumption (eating every day, pieces p/week)  Results favour intervention | Townsend Deprivation Score  No preferential impact | Weak |
| **Friel-1999 (Ireland)(10)** | | | | | | | |
| Design: quasi-RCT  Aim: To assess change in children’s behaviour resulting from the pilot intervention | Irish schoolchildren aged 8-10 years from 8 schools (n=812) | Diet | Education programme comprising 20 sessions over 10 weeks | Recipients: School children  Components (n=5)  1. Lesson plans for teachers  2. Activity worksheets for pupils  3. Home team pack  4. Teacher training  5. Aerobic exercise regime | Food pairing questionnaire score  Results favour intervention | Area level deprivation  Likely to widen inequalities | 2 |
| **Haerens-2007 (Belgium)(11)** | | | | | | | |
| Design: Cluster-RCT  Aim: To investigate the short term impact of a computer tailored dietary fat intake intervention in adolescents | 7^th^ grade pupils in 10 schools from two cities in Belgium (n=304) | Diet | Theory based computer tailored dietary fat intake intervention | Recipients: School children  Components (n=1)  1. Tailored CD-ROM containing computer tailored dietary fat intervention | Dietary fat intake  No difference | Education level  No preferential impact | 4 |
| **Havas-1998 (USA)(12)** | | | | | | | |
| Design: RCT  Aim: To increase fruit and vegetable consumption among women served by WIC programmes | Women attending Supplemental Nutrition Program for Women, Infants and Children, 16 sites in Baltimore City and 6 in Maryland (n=3122) | Diet | The intervention consisted of nutrition sessions conducted by peer educators; printed materials and visual reminders and direct mail. All designed to help participants progress through the stages of change. | Recipients: Low income mothers  Components (n=12)  *Nutrition education*  1. Brief messages at time of enrolment  2. Group discussion session  *Direct mail*  3. Tip sheets  4. Clue cards  5. Tailored letters  *Printed materials*  6. Photonovella  7. Booklet of recipes  8. Children’s activities  9. Videotape for children  10. Refrigerator magnet  11. Calendar sheets and stickers  12. Fruit and vegetable posters | Fruit and vegetable consumption  Results favour intervention | Education level  Likely to widen inequalities | 5 |
| **Havas-2003 (USA)(13)** | | | | | | | |
| Design: RCT  Aim: To decrease percent of calories derived from fat and to increase fruit, vegetable and fibre intake among low-income women served by the WIC programme in Maryland | Women attending Supplemental Nutrition Program for Women, Infants and Children, 10 sites in Baltimore City and Maryland (n=2066) | Diet | Intervention participants were invited to five interactive nutrition sessions and sent written materials | Recipients: Low income mothers  Components (n=18)  1. 5 minute video with enthusiastic previous participants  2. Food for Life brochure  3. Individualised feedback on baseline data  4. Kick off fair  5. 4x45 minute workshops  6. Newsletters  7. Mail packets  8. Personalised invitations  9. Behaviour re-enforcing incentives  10. Phone calls  11. Group discussion on favourite foods and methods to cook  12. Product comparisons  13. Food demonstration at workshop  14. Healthy Meal  15. Tip sheets  16. Brief individualised counselling  17. Interactive cooking demonstration at fair  18. Free bag of food | % calories from fat, fruit and vegetable servings, fibre intake  Results favour intervention | Education level  Likely to widen inequalities | 5 |
| **Hobin-2014 (Canada)(14)** | | | | | | | |
| Design: Longitudinal study  Aim: To examine longitudinal changes in and factors associated with the PA trajectories of adolescents in Manitoba in secondary school | 31 schools in Manitoba Province, Canada (n=447) | PA | Province wide PE policy extending secondary school graduation requirements. | Recipients: School children  Components (n=3)  1. ”Core component” – In class study of health  2. “Flexible component” – exploring areas of interest/specialisation  3. “PA practicum” – PA participation in and out of class | Minutes MVPA per day  Results favour control | School neighbourhood disadvantage  Likely to reduce inequalities | Moderate |
| **Jeffery-1998 (USA)(15)** | | | | | | | |
| Design: RCT  Aim: To examine whether weight gain with age can be prevented using a low intensity intervention | 20-45 year old women in 4 local health departments in Minneapolis/St Paul, Minnesota metropolitan area (n=593) | Diet and PA | Monthly newsletter and face to face health education plus lottery to encourage participation | Recipients: Adults aged 20-45  Components (n=4)  1.Monthly newsletter  2. Postcard to encourage reflection on healthy habits  3. Face to face education programme  4. Lottery to incentivise participation | % energy from fat; Fruit servings p/day; Vegetable servings p/day  No difference  PA score; walks p/week  PA: No difference | Income  Diet: No preferential impact  PA: No preferential impact | 3 |
| **Lowe-2004 (UK)(16)** | | | | | | | |
| Design: Before-after study  Aim: To assess how a peer modelling and rewards based intervention changes children’s consumption of fruit and vegetables | 4 – 11 year old children from 1 school in Wales and 2 in England (n=402) | Diet | Video adventures that include the Food Dudes and small rewards for consuming fruit and vegetables | Recipients: School children  Components (n=5)  1. Peer modelling videos  2. Rewards for healthy eating  3. Letters from cartoon characters to children  4. Homepacks to encourage healthy eating at home  5. Home sticker cards | Snack time fruit consumption; snack time vegetable consumption  Results favour intervention | % Free School Meals  Likely to widen inequalities | 3 |
| **Millett-2011 (UK)(17)** | | | | | | | |
| Design: Repeat cross-sectional  Aim: To evaluate the impact of the national salt strategy in England | Representative sample of adults living in England (n=1668) | Diet | National salt reduction strategy | Recipients: Adults in UK  Components (n=3)  Voluntary agreements with food industry to…  1. reduce salt content in processed foods  2. improve food labelling  3. implement public awareness campaigns to change individual behaviour | Daily salt intake  Results favour intervention | Social class  No preferential impact | 3 |
| **Moore-2014 (UK)(18)** | | | | | | | |
| Design: Cluster RCT  Aim: To examine universal provision of breakfast on socioeconomic inequalities in children’s dietary behaviour | Children aged 9-11 from 111 schools in Wales  (Baseline n=4350; Follow up n=4472) | Diet | Primary school free breakfast initiative | Recipients: School children  Components (n=1)  1. Provision of breakfast at school | Healthy items at breakfast; Unhealthy items at home  No difference | Free School Meal  No preferential impact | Moderate |
| **Reynolds-2000 (USA)(19)** | | | | | | | |
| Design: Cluster RCT  Aim: To evaluate the effects of a school based dietary intervention to increase fruit and vegetable intake | Fourth grade children from 28 elementary schools (n=1698 families) | Diet | Three component intervention consisting of classroom component, parent component and food service component | Recipients: School children  Components (n=9)  *Classroom component*  1. Lessons in school  *Parent component*  2. Homework assignments  3. Brochures  4. Information evening  5. Recipes  6. Items to trigger behaviour  *Food service component*  7. Half day training  8. Cafeteria tasks and ratings  9. Visits from nutritionists | Fruit consumption; Vegetable consumption  Results favour intervention | Household income  No preferential impact | 3 |
| **Robroek-2012 (Netherlands)(20)** | | | | | | | |
| Design: Cluster-RCT  Aim: To evaluate cost-effectiveness of a long term workplace health promotion programme on PA and diet | 76 departments within 6 workplaces (healthcare organisations, commercial services and executive branch of government)  (n=924) | Diet and PA | Additional website functionalities includes action-oriented feedback, self-monitoring, possibility to ask questions and monthly email messages | Recipients: Workplace volunteers  Components (n=5)  1.Computer tailored advice on self-report PA and diet  2. Online self-monitors on fruit and veg intake, PA and weight to monitor progress  3. Food frequency questionnaire assessing saturated fat intake  4. Possibility to submit questions to health professionals  5. Monthly email messages | Fruit intake; vegetable intake  Diet: No difference  PA: No difference | Education level  Diet: No preferential impact  PA: No preferential impact | Moderate to strong |
| **Rosenkranz-2010 (USA)(21)** | | | | | | | |
| Design: Cluster RCT  Aim: To evaluate an intervention designed to prevent obesity by modifying Girl Scout troop meeting environments | 7 Girl Scout Troops consisting of girls in 4^th^ and 5^th^ grades (n=76) | Diet and PA | Environmental intervention in girl scout troops based on three components of social cognitive theory: Role modelling; skill building; enhancement of self-efficacy and proxy efficacy; and reinforcement of behaviour | Recipients: Girl Scouts  Components (n=11)  *Interactive educational curriculum*  1. Discussion of target behaviours  2. Worksheet for goal setting and monitoring  3. Physically active recreation session  4. Fruit and Veg snack recipe preparation  5. Family meal role-playing  *Troop meeting policies implemented by troop leaders*  6. Providing 15 minutes per meeting for physically active recreation  7. Troop leaders participation in PA with girls  8. Provision of a fruit and veg snack prepared by girls  9. Troop leaders eating fruit and veg snack with girls  10. Troop leaders verbally promoting PA, Fruit and veg in meetings and home  11. Prohibition of SSB, candy and TV watching during meetings | FV daily consumption; SSB daily consumption  Diet: Results favour intervention  PA: Results favour intervention | Socioeconomic status  PA: No preferential impact | Strong |
| **Simon-2008 (France)(22)** | | | | | | | |
| Design: Cluster RCT  Aim: To assess a PA intervention in adolescents | 8 middle schools in Eastern France  (n=954) | PA | The intervention was designed to promote PA by changing attitudes through debates and attractive activities and by providing social support and environmental changes encouraging PA | Recipients: School children  Components (n=7)  1.Educational component focusing on PA and sedentary behaviour  2. PA opportunities at lunchtime  3. PA opportunities during breaks  4. PA opportunities after school  5. Sporting events  6. Cycle to school days  7. Regular meetings with parents and educators | Supervised leisure PA; Active home-school commuting  No difference | Occupation category  No preferential impact | Strong |
| **Smith-1997 (Australia)(23)** | | | | | | | |
| Design: RCT  Aim: To estimate population uptake and effectiveness of dietary behaviour change counselling in higher and lower socio-economic status groups | Randomly selected population sample in Adelaide (n=479) | Diet | The intervention was based on behaviour change techniques and social learning theories and included a one time counselling session with follow up after 3 months including individualised feedback and advice | Recipients: Adults  Components (n=3)  1.Dietary counselling session  2. Booklet containing cholesterol test result  3. Self-monitoring charts | Food group and nutrient consumption  No difference | Occupational prestige  No preferential impact | 4 |
| **Stables-2002 (USA)(24)** | | | | | | | |
| Design: Repeat cross sectional  Aim: To assess population-based changes in vegetable and fruit consumption and psychosocial correlates | Representative sample of US adults (Baseline – n=2755; Follow up – n=2544) | Diet | The 5 a day for better health program is a nutrition education campaign designed to increase awareness of the need to consume more FV | Recipients: Adults  Components (n=1)  1. 5-a-day for better health program (nutrition education) | No difference | Poverty Index Ratio  No preferential impact | 2 |
| **Sturm-2010 (USA)(25)** | | | | | | | |
| Design: Cross-sectional with unexposed group  Aim: To examine whether small taxes are likley to change consumption and weight gain or whether larger taxes would be needed | Nationally representative US kindergartners (n=7403) | Diet | State level soda taxes | Recipients: School children  Components (n=1)  1. Taxation of soft drinks | SSB consumption  No difference | Family income  Likely to reduce inequalities | Moderate |
| **Taber-2013 (USA)(26)** | | | | | | | |
| Design: Cross-sectional with unexposed group  Aim: To determine if state laws with stricter school meal nutrition standards are inversely associated with adolescent weight status | 8^th^ grade students in 40 US states (n=4870) | Diet | State laws governing school meal nutrition standards | Recipients: School children  Components (n=1)  1. State laws governing school meal nutrition standards | Fast food and confectionary intake  No difference | Free school meal provision  No preferential impact | Moderate |
| **Tak-2008 & 2010 (Netherlands) (27, 28)** | | | | | | | |
| Design: Longitudinal study  Aim: To evaluate long term effects of a Dutch primary school based intervention providing free fruit and vegetables | 9-10 year old children in 44 Dutch schools in 5 cities (n=771) | Diet | Free fruit and veg in schools improving the availability, accessibility and exposure to fruit and veg in schools | Recipients: School children  Components (n=4)  1. Provision of a portion of free fruit or veg twice a week  2. Optional school curriculum to increase knowledge and skills  3. Provision of a lunchbox  4. Provision of a calendar for teachers indicating when FV would be provided | Results favour intervention | Parental education  No preferential impact | Strong |
| **Toftager-2014 (Denmark)(29)** | | | | | | | |
| Design: Cluster RCT  Aim: To assess the effectiveness of a multi-component environmental school-based intervention to reduce the age-related decline in PA among adolescents | 14 schools in Southern Denmark (n=1348) | PA | Intervention changing the physical and organisational environment of the schools | Recipients: School children  Components (n=14)  1. Upgrading existing outdoor areas at the school for PA  2. Building playgrounds designed for adolescents  3. Improving safety for active transport to and from school  4. Establishing an afterschool fitness program  5. Implementing school PA policy  6. Educating teachers as ‘kick starters’ who facilitate PA during breaks  7. School play patrol  8. Mandatory outdoor recess and/or free access to gym/sports hall  9. School traffic patrol  10. Safe cycling training for students  11. School project/theme week once a year | FV bought from home; Unhealthy food bought from home  No difference | Household income  No preferential impact | Moderate |
| ^a^ Quality assessment as assigned in the parent systematic reviews  MVPA = moderate to vigorous physical activity; FV = Fruit and vegetable; SSB = Sugar Sweetened Beverage | | | | | | | |

**References**

1. Bere E, Veierød MB, Klepp K-I. The Norwegian School Fruit Programme: evaluating paid vs. no-cost subscriptions. Preventive medicine. 2005;41(2):463-70.

2. Bere E, Hilsen M, Klepp K-I. Effect of the nationwide free school fruit scheme in Norway. British Journal of Nutrition. 2010;104(4):589-94.

3. Brownson RC, Smith CA, Pratt M, Mack NE, Jackson-Thompson J, Dean CG, et al. Preventing cardiovascular disease through community-based risk reduction: the Bootheel Heart Health Project. American Journal of Public Health. 1996;86(2):206-13.

4. Campbell MK, Demark-Wahnefried W, Symons M, Kalsbeek WD, Dodds J, Cowan A, et al. Fruit and vegetable consumption and prevention of cancer: the Black Churches United for Better Health project. American journal of public health. 1999;89(9):1390-6.

5. Coronini-Cronberg S, Millett C, Laverty AA, Webb E. The impact of a free older persons’ bus pass on active travel and regular walking in England. American Journal of Public Health. 2012;102(11):2141-8.

6. Cullen KW, Watson K, Zakeri I. Improvements in middle school student dietary intake after implementation of the Texas Public School Nutrition Policy. American Journal of Public Health. 2008;98(1):111-7.

7. Cullen KW, Watson KB, Fithian AR. The impact of school socioeconomic status on student lunch consumption after implementation of the Texas Public School Nutrition Policy. Journal of School Health. 2009;79(11):525-31.

8. Mendoza JA, Watson K, Cullen KW. Change in dietary energy density after implementation of the Texas Public School Nutrition Policy. Journal of the American Dietetic Association. 2010;110(3):434-40.

9. Fogarty A, Antoniak M, Venn A, Davies L, Goodwin A, Salfield N, et al. Does participation in a population-based dietary intervention scheme have a lasting impact on fruit intake in young children? International Journal of Epidemiology. 2007;36(5):1080-5.

10. Friel S, Kelleher C, Campbell P, Nolan G. Evaluation of the nutrition education at primary school (NEAPS) programme. Public health nutrition. 1999;2(4):549-55.

11. Haerens L, Deforche B, Maes L, Brug J, Vandelanotte C, De Bourdeaudhuij I. A computer-tailored dietary fat intake intervention for adolescents: results of a randomized controlled trial. Annals of Behavioral Medicine. 2007;34(3):253-62.

12. Havas S, Anliker J, Damron D, Langenberg P, Ballesteros M, Feldman R. Final results of the Maryland WIC 5-a-day promotion program. American journal of public health. 1998;88(8):1161-7.

13. Havas S, Anliker J, Greenberg D, Block G, Block T, Blik C, et al. Final results of the Maryland WIC food for life program. Preventive Medicine. 2003;37(5):406-16.

14. Hobin E, So J, Rosella L, Comte M, Manske S, McGavock J. Trajectories of objectively measured physical activity among secondary students in Canada in the context of a province-wide physical education policy: a longitudinal analysis. Journal of Obesity. 2014;2014.

15. Jeffery R, French S. Preventing weight gain in adults: design, methods and one year results from the Pound of Prevention study. International Journal of Obesity. 1997;21(6):457-64.

16. Lowe CF, Horne PJ, Tapper K, Bowdery M, Egerton C. Effects of a peer modelling and rewards-based intervention to increase fruit and vegetable consumption in children. European journal of clinical nutrition. 2004;58(3):510-22.

17. Millett C, Laverty AA, Stylianou N, Bibbins-Domingo K, Pape UJ. Impacts of a national strategy to reduce population salt intake in England: serial cross sectional study. PLoS One. 2012;7(1):e29836.

18. Moore GF, Murphy S, Chaplin K, Lyons RA, Atkinson M, Moore L. Impacts of the Primary School Free Breakfast Initiative on socio-economic inequalities in breakfast consumption among 9–11-year-old schoolchildren in Wales. Public health nutrition. 2014;17(6):1280-9.

19. Reynolds KD, Franklin FA, Binkley D, Raczynski JM, Harrington KF, Kirk KA, et al. Increasing the fruit and vegetable consumption of fourth-graders: results from the high 5 project. Preventive medicine. 2000;30(4):309-19.

20. Robroek SJ, Polinder S, Bredt FJ, Burdorf A. Cost-effectiveness of a long-term Internet-delivered worksite health promotion programme on physical activity and nutrition: a cluster randomized controlled trial. Health education research. 2012;27(3):399-410.

21. Rosenkranz RR, Behrens TK, Dzewaltowski DA. A group-randomized controlled trial for health promotion in Girl Scouts: healthier troops in a SNAP (Scouting Nutrition & Activity Program). BMC public health. 2010;10:1-13.

22. Simon C, Schweitzer B, Oujaa M, Wagner A, Arveiler D, Triby E, et al. Successful overweight prevention in adolescents by increasing physical activity: a 4-year randomized controlled intervention. International journal of obesity. 2008;32(10):1489-98.

23. Smith AM, Owen N, Baghurst KI. Influence of socioeconomic status on the effectiveness of dietary counselling in healthy volunteers. Journal of Nutrition Education. 1997;29(1):27-35.

24. Stables GJ, Subar AF, Patterson BH, Dodd K, Heimendinger J, Van Duyn MAS, et al. Changes in vegetable and fruit consumption and awareness among US adults: results of the 1991 and 1997 5 A Day for Better Health Program surveys. Journal of the American Dietetic Association. 2002;102(6):809-17.

25. Sturm R, Powell LM, Chriqui JF, Chaloupka FJ. Soda taxes, soft drink consumption, and children’s body mass index. Health Affairs. 2010;29(5):1052-8.

26. Taber DR, Chriqui JF, Powell L, Chaloupka FJ. Association between state laws governing school meal nutrition content and student weight status: implications for new USDA school meal standards. JAMA pediatrics. 2013;167(6):513-9.

27. Tak N, Te Velde S, Singh A, Brug J. The effects of a fruit and vegetable promotion intervention on unhealthy snacks during mid‐morning school breaks: results of the Dutch Schoolgruiten Project. Journal of human nutrition and dietetics. 2010;23(6):609-15.

28. Tak NI, Te Velde SJ, Brug J. Long-term effects of the Dutch Schoolgruiten Project–promoting fruit and vegetable consumption among primary-school children. Public health nutrition. 2009;12(8):1213-23.

29. Toftager M, Christiansen LB, Ersbøll AK, Kristensen PL, Due P, Troelsen J. Intervention effects on adolescent physical activity in the multicomponent SPACE study: a cluster randomized controlled trial. PLoS One. 2014;9(6):e99369.
